# Demography and Clinical Profile of Heatstroke Patients

**DOI:** 10.7759/cureus.81852

**Published:** 2025-04-07

**Authors:** Aji Antony, Jayesh Kumar P, Geetha P, Abdul Majeed K, Jayachandran N.V, Mohammed Shaan, Mridul Kumar K, Soopy Kayanaduth

**Affiliations:** 1 Internal Medicine, Government Medical College, Kozhikode, Kozhikode, IND

**Keywords:** altered sensorium, climate change, heat stroke, multiorgan dysfunction syndrome, seizures

## Abstract

Background: Heatstroke, characterized by a significant rise in body temperature and central nervous system dysfunction, poses a severe health risk, particularly during heatwaves.

Aim: This study aims to describe the epidemiological and clinical profile of heatstroke patients admitted to our centre during a heatwave period.

Results: A total of nine cases were recorded during the study period. All patients were male, aged between 35 and 63 years, with the majority working in occupations involving direct exposure to sunlight. The majority of the patients presented with seizures and altered sensorium, with temperatures exceeding 105°F in most cases. Blood investigations revealed leukocytosis, thrombocytopenia, and multi-organ dysfunction. Creatine phosphokinase (CPK) levels were invariably high in all patients, and arterial blood gas analysis revealed high-anion-gap metabolic acidosis (HAGMA). Treatment measures given include cooling measures, inotropes, and supportive care. The case fatality rate was 55%, highlighting the severity of the cases.

Conclusions: As global temperatures rise and heatwaves become more frequent due to climate change, heatstroke is progressively emerging as a major public health issue. Risk factors such as occupational sun exposure, comorbidities, and extreme temperatures contribute to heatstroke incidence and severity. Urgent public health strategies are needed to mitigate heatwave impacts and protect vulnerable populations. Further research into novel treatment approaches may improve outcomes for heatstroke patients.

## Introduction

Heatstroke is the most dangerous condition in a spectrum of illnesses ranging from heat exhaustion to heatstroke, in which a common finding is hyperthermia (i.e., the rise in core body temperature when heat accumulation overrides heat dissipation during exercise or exposure to environmental heat stress [1]. The incidence of heatstroke varies depending on geographical location, climate patterns, and population characteristics. Recently, there has been an increasing trend of heat-related events such as heatstroke. In India, the incidence of heatstroke is also rising, and there is growing concern about the same. The number of heatstroke-related deaths in India surged past 2,500 in 2015, representing a fivefold increase since 2001. However, recent epidemiological data on heat-related mortality remain limited [2]. The high-risk populations for heatstroke include those in the extremes of age, athletes, and individuals with premorbid medical illnesses. Similarly, certain occupational groups, such as those involved in agricultural and constructional activities and military personnel, have an increased incidence of heatstroke [3]. The two types of heatstroke described in the literature are as follows: classic heatstroke and exertional heatstroke [4]. The pathogenesis of heatstroke involves a systemic inflammatory response resulting in multiorgan dysfunction [5].

Clinical manifestations include elevated body temperature (>40°C or 104°F), altered sensorium, hot and dry skin (in classic heatstroke) or profuse sweating (in exertional heatstroke), nausea, and vomiting. These often culminate in seizures, multi-organ dysfunction, and death if not treated [6]. The three clinical phases of heatstroke include the neurologic acute phase, a hematologic-enzymatic phase, and a late renal-hepatic phase. Identification of the disease during the acute neurologic phase is essential for prompt treatment and a better prognosis. The most important objective in the management of heatstroke is the alleviation of hyperthermia. Though there are various methods of cooling, immersion in cold water is the accepted method of choice. Institution of cooling measures in the early stage of the disease often prevents the multiorgan dysfunction. However, once organ failure sets in, supportive care, depending on the organ system involved, should be initiated [7-9].

Heatstroke has been recognized as a rising public health concern globally due to the changing climate. India has also reported a significant rise in heatstroke cases in the last few years. Despite the above concern, there is a paucity of data on the epidemiology and clinical features of heatstroke. Hence, this study aims to describe the epidemiological and clinical features of heatstroke patients admitted to our hospital over the past year.

## Materials and methods

This study is a descriptive case series of patients with heatstroke admitted to Government Medical College, Kozhikode, India, during the period from March 2024 to May 2024. Ethical approval was obtained from the institutional ethics committee before the study commenced. Heatstroke is defined as “a form of hyperthermia associated with a systemic inflammatory response, resulting in multiorgan dysfunction syndrome with predominant encephalopathy” [3]. Due to the nonspecific nature of early symptoms, diagnosis relied on a thorough clinical assessment, incorporating factors such as physical exertion, extreme hyperthermia, central nervous system disturbances (e.g., coma, stupor, delirium), hematologic and enzymatic abnormalities (e.g., clotting dysfunction, rhabdomyolysis), and clinical manifestations (e.g., seizures, shock, brain edema). It remains primarily a clinical diagnosis supported by laboratory investigations. All clinically diagnosed cases of heatstroke were included in the study, while potential alternative diagnoses were systematically excluded. A time-bound sampling method was used, and all heatstroke patients admitted during the study period were included. Case sheets of the admitted patients were retrieved, and confidentiality of patient identity was maintained. Data were anonymized by removing identifiers.

A structured case record form was employed to document demographic details, clinical findings, and outcomes. Demographic information collected included age (in years), sex, date of presentation, district of residence, and occupation. Daily heat exposure and specific heat exposure on the day of the incident were also documented. The clinical profile included symptoms, body temperature (°F), blood pressure (mmHg), pulse rate (per minute), oxygen saturation (SpO₂), and a detailed nervous system examination, including the Glasgow Coma Scale (GCS), pupil size and reactivity, reflexes, muscle tone, and plantar response. Respiratory system findings were also noted. In addition, several laboratory parameters were recorded, including total leukocyte count (per/mm³), hemoglobin (g/dL), platelet count (per/mm³), international normalized ratio (INR), serum creatinine (mg/dL), serum electrolytes (sodium/potassium, mmol/L), random blood sugar (mg/dL), serum glutamic pyruvic transaminase (U/L), serum glutamic oxaloacetic transaminase (U/L), bilirubin (mg/dL), arterial blood gas analysis, creatinine phosphokinase (U/L), and quantitative troponin I (ng/mL). Imaging findings from echocardiography, computed tomography (CT) of the head, and electrocardiography (ECG) were also documented. The treatment administered to each patient and the outcomes were recorded. Statistical analysis was performed by entering the collected data into an Excel spreadsheet, followed by frequency analysis of the study variables using proportions and percentages.

## Results

A total of nine cases were reported during the study period between March and May 2024. The patients, aged between 35 and 63 years, were primarily engaged in outdoor or labor-intensive occupations. The majority of cases were from Kozhikode, with a few from Malappuram. Daily heat exposure ranged from eight to 12 hours, and on the day of the heatstroke event, exposure duration varied: six patients had four to six hours of heat exposure, and two home-bound patients developed symptoms later in the evening (around 6-8 PM). Table 1 outlines the clinical profile, diagnostic findings, treatment given, and outcomes of the nine heatstroke patients. All patients presented with elevated body temperatures ranging from 100°F to 106°F. The predominant symptoms observed were seizures and altered sensorium. Blood pressure readings varied, with most patients exhibiting hypotension.

**Table 1:** Clinical profile of the nine heatstroke patients Abbreviations: GCS = Glasgow Coma Scale - E (Eye Opening Response), V (Verbal Response), M (Motor Response)/ FiO2 = Fraction of inspired Oxygen

Number	Symptoms	Temperature (°F)	Blood Pressure (BP) in Millimeters of Mercury (mmHg)	Pulse Rate (PR) per Minute	Oxygen Saturation (SpO₂)	Nervous System Examination (GCS, Pupils, Reflexes, Muscle Tone, Plantar Response)	Respiratory System Examination
1	Fell unconscious while painting, followed by seizures	105.6	85/65	112	86%	Drowsy (GCS: E1V1M5); Pupils: both 2 millimeters, sluggishly reactive; Dolls eye reflex present; Muscle Tone: normal; Plantar response: both flexor	Bilateral conducted sounds
2	Fell unconscious, followed by seizures	106	80/50	153	81%	Drowsy (GCS: E1V1M5); Pupils: both 2 millimeters, non-reactive; Dolls eye reflex present; Muscle Tone: normal; Plantar response: both flexor	Bilateral coarse crepitations
3	Known psychiatric illness, on antipsychotics, fell unconscious, followed by seizures	100	100/70	122	99%	Drowsy (GCS: E1V1M5); Pupils: both 2 millimeters, reactive; Dolls eye reflex present; Muscle Tone: normal; Plantar response: both mute	Normal vesicular breath sounds
4	Fell unconscious, followed by seizures	102	120/80	52	99%	Drowsy (GCS: E2V2M5); Pupils: both 1 millimeter, reactive; Dolls eye reflex present; Muscle Tone: normal; Plantar response: both flexor	Normal vesicular breath sounds
5	Found unresponsive in workplace with high temperature	107	100/70	132	85%	Drowsy (GCS: E2V2M5); Pupils: both 2 millimeters, sluggishly reactive; Dolls eye reflex present; Muscle Tone: normal; Plantar response: both mute	Bilateral coarse crepitations
6	Psychiatric illness, on antipsychotics, fell unconscious, followed by seizures	106	107/65	160	98%	Drowsy (GCS: E3V1M4); Pupils: both 2-3 millimeters, non-reactive; Dolls eye reflex present; Muscle Tone: cogwheel rigidity; Plantar response: both mute	Bilateral coarse crepitations
7	History of evacuation of an epidural hematoma two years back, on antiepileptic medications (Brivaracetam and Sodium Valproate), alcoholic, history of heavy exertion and alcohol intake one day prior, found unresponsive with signs of postictal state	100	100/60	120	98%	Drowsy (GCS: E2V1M5); Pupils: both 2 millimeters, sluggishly reactive; Dolls eye reflex present; Muscle Tone: normal; Plantar response: both mute	Normal vesicular breath sounds
8	Known case of hypertension and depression on clomipramine, presented with fatigue, intubated due to worsening neurological status	108	170/114	110	98% (on 30% fraction of inspired oxygen)	Intubated (GCS: E1VTM2); Pupils: both 2 millimeters, sluggishly reactive; Dolls eye reflex present; Muscle Tone: normal; Plantar response: extensor	Normal vesicular breath sounds
9	Found unresponsive in workplace	106	114/60	153	81%	Drowsy (GCS: E1V1M1); Pupils: both 2 millimeters, non-reactive; Dolls eye reflex present; Muscle Tone: normal; Plantar response: both flexor	Bilateral coarse crepitations

The laboratory investigations of the nine heatstroke patients are shown in Table 2.

**Table 2:** Blood investigations of the nine heatstroke patients

Number	Total Leukocyte Count (per/mm^3^)	Hemoglobin (g/dL)	Platelet Count (per/mm^3^)	International Normalized Ratio (INR)	Serum Creatinine (mg/dL)	Serum Electrolytes (Na / K, mmol/L)	Random Blood Sugar (mg/dL)	Serum Glutamic Pyruvic Transaminase (U/L)	Serum Glutamic Oxaloacetic Transaminase (U/L)	Bilirubin (mg/dL)	Arterial Blood Gas Analysis	Creatinine Phosphokinase (U/L)	Quantitative Troponin I (ng/mL)
1	11,100	12.6	118,000	1.36	2.4	122 / 3.9	126	85	47	2.3	High Anion Gap Metabolic Acidosis	1077	0.8
2	14,500	14	101,000	1.36	2.4	122 / 3.9	126	254	107	0.8	High Anion Gap Metabolic Acidosis	1034	0.8
3	21,600	11.6	264,000	1.16	2	138 / 3.4	278	40	62	0.6	High Anion Gap Metabolic Acidosis	1155	0.074
4	14,200	12.4	176,000	1.33	0.8	115 / 3.0	156	14	33	0.6	High Anion Gap Metabolic Acidosis	1079	Negative
5	18,200	13.7	112,000	2.7	2.8	136 / 3.9	182	83	98	0.7	High Anion Gap Metabolic Acidosis	3073	5.2
6	15,610	13.9	112,000	1.51	0.9	126 / 3.0	193	68	67	0.5	High Anion Gap Metabolic Acidosis	3300	1.1
7	12,400	16.1	96,000	1.5	3.3	147 / 3.4	129	715	2170	1.9	High Anion Gap Metabolic Acidosis	42,000	Negative
8	9,800	13.9	84,000	1.68	4.4	128 / 2.2	139	53	210	2	High Anion Gap Metabolic Acidosis	8560	0.28
9	31,500	15.3	84,000	2.53	2.1	131 / 3.7	149	62	92	0.8	High Anion Gap Metabolic Acidosis	5212	1.6
Normal range	4000-11,000 cells/mm^3^	14-17 g/dL (men); 12-15 g/dL (women)	150,000-400,000 cells/mm^3^	Less than 1.1	0.7-1.3 mg/dL (men); 0.6-1.1 mg/dL (women)	Na -135-145 mmol/L / K- 3.5-5 mmol/L	80-200mg/dL	5-40 U/L	5-40 U/L	0.1-1.2 mg/dL	Normal pH = 7.35-7.45	10-120 U/L	<0.03 ng/mL

The total leukocyte count ranged from 9,800 to 31,500 cells/mm³, with a mean of 16,545 cells/mm³. Hemoglobin levels ranged from 11.6 to 16.1 g/dL. There was thrombocytopenia in most patients. Liver function tests were deranged in almost all patients. The mean creatinine level was 2.34 mg/dL. Creatinine phosphokinase (CPK) showed a wide range from 1,034 to 42,000 U/L, with a mean of 7,388 U/L, indicating rhabdomyolysis.

Table 3 outlines the imaging findings, treatment given, and outcomes of the nine heatstroke patients. Echocardiography indicated left ventricular dysfunction in four patients. While most CT scans of the head showed no significant abnormalities, one patient exhibited diffuse brain edema. Treatment involved various interventions, including cooling measures, inotropes, anti-seizure medications, hemodialysis, and mechanical ventilation, tailored to individual patient needs. The case fatality rate stood at 55%, with five out of nine patients succumbing to the condition, four of whom passed away within 24 hours of presentation.

**Table 3:** Radiological investigations, course, and outcome of the nine heatstroke patients

Number	Computed Tomography (CT) Imaging of Brain	Electrocardiography (ECG) Findings	Echocardiography (ECHO) Findings	Course and Outcome
1	Normal	Sinus tachycardia	Occasional B lines, Good left ventricular function	Patient electively intubated and succumbed 14 hours later.
2	Normal	Diffuse ST depression, aVR ST elevation	Global left ventricular hypokinesia	Patient electively intubated. Started on intravenous (IV) fluids and supportive measures but succumbed 16 hours later.
3	Normal	T wave inversions	Normal	Started on antiepileptics, IV fluids, and supportive measures. Patient extubated on day 2 and recovered.
4	Diffuse brain edema	Normal	Normal	Developed progressive worsening of consciousness. Electively intubated. Developed one episode of hematemesis (vomiting of blood) after intubation. Started on antiepileptics, brain edema management, and hyponatremia correction. Patient extubated on day 2 of admission and recovered.
5	Normal	Diffuse ST depression, aVR ST elevation	Global left ventricular hypokinesia	Developed progressive worsening of consciousness and was electively intubated. Developed one episode of hematemesis post intubation. Started on antiepileptics, IV fluids, fresh frozen plasma (FFP), and supportive measures. Patient expired 12 hours after admission.
6	Age related brain atrophy	Right Bundle Branch Block (RBBB) with Right Axis Deviation (RAD)	Normal	Started on antiepileptics, IV fluids, and supportive measures. Patient eventually recovered after 15 days of intensive care unit (ICU) care.
7	Craniotomy defect, rest normal	Diffuse ST depression, aVR ST elevation	Normal	Started on antiepileptics, IV fluids, and supportive measures. Patient developed progressive acute kidney injury (AKI), supported with hemodialysis (HD), but progressively worsened and succumbed.
8	Normal	Diffuse ST depression, aVR ST elevation	Global left ventricular hypokinesia	Intubated from outside center. Started on IV fluids and supportive measures. Patient eventually recovered.
9	Normal	Diffuse ST depression, aVR ST elevation	Global left ventricular hypokinesia	Treated with supportive measures but succumbed one hour later.

## Discussion

This case series demonstrates the demography, clinical features, and outcomes of heatstroke patients. There were nine heatstroke patients admitted during the study period. The predominance of male patients aligns with numerous studies indicating a higher frequency of heat-related illnesses among males, often linked to patterns of occupational exposure. The age range of 35-63 is in line with previous findings, suggesting that heat-related illnesses can affect adults across a wide range of ages [1-3]. Occupations involving direct sun exposure, such as painting and construction work, reflect well-known risk factors associated with heat-related illnesses, emphasizing the importance of implementing occupational health measures to mitigate these risks. The presence of psychiatric illness and alcoholism as comorbidities corresponds with existing research, highlighting the significance of underlying health conditions in exacerbating susceptibility to heat-related complications [4,5]. The predominant symptoms of seizures and altered sensorium are consistent with severe manifestations of heat-related illnesses reported in the literature, indicating the potential severity of the observed cases. The case fatality rate of 55% is notably higher than reported averages, underscoring the severity of the cases and potentially indicating the need for improved preventive strategies and early interventions in high-risk populations [6,7]. Novel treatment approaches are being explored in animal models and early clinical studies, hinting at potential future therapies. These include using allopurinol, a xanthine oxidase inhibitor, to reduce portal lipopolysaccharide levels by safeguarding tight cell-to-cell junctions. Additionally, recombinant activated protein C is being investigated to mitigate inflammation and the dysfunctional coagulation cascade. Other treatments under study involve type III antithrombin concentrate and recombinant soluble thrombomodulin-alpha for treating disseminated intravascular coagulation (DIC), alongside serine proteases to inhibit pancreatic enzyme activity in the intestinal lumen, thus reducing systemic inflammatory markers [10-12].

Heatwaves are increasingly impacting health, especially among those with occupational sun exposure in Kerala, mirroring broader trends in India and other heatwave-prone regions. Studies show heatwaves raise morbidity, mortality, and disease burden, emphasizing the need for comprehensive public health planning [13,14]. Rising heatwave frequency, intensity, and duration, particularly by the century's end, pose escalating risks. Human activities contribute significantly to severe heatwaves, with humidity exacerbating health risks [15]. Enhanced public health communication is vital to mitigate heatwave impacts. These findings stress the urgent need for robust public health strategies to protect vulnerable populations during extreme weather events.

The main limitation of this study is the small sample size, with only nine patients included over a short period. This limits the generalizability and statistical strength of the findings. Larger, longer-term studies are needed to validate and expand upon these observations.

## Conclusions

This study aims to acquaint clinicians with the epidemiological and clinical characteristics of patients suffering from heatstroke. Detecting a case of heatstroke requires a high level of suspicion. Prompt implementation of treatment protocols, including cooling measures, is crucial in preventing organ damage and fatalities. Preventing heatstroke is more effective and less challenging than treating it afterward. Therefore, public health interventions should be implemented to alleviate the impacts of heatwaves, particularly among high-risk populations. Additionally, further research into the pathogenesis and treatment strategies of heatstroke is warranted.
